# *p66Shc* gene expression in peripheral blood mononuclear cells and progression of diabetic complications

**DOI:** 10.1186/s12933-018-0660-9

**Published:** 2018-01-17

**Authors:** Gian Paolo Fadini, Mattia Albiero, Benedetta Maria Bonora, Nicol Poncina, Saula Vigili de Kreutzenberg, Angelo Avogaro

**Affiliations:** 1 Department of Medicine, University of Padova, Via Giustiniani 2, 35100 Padua, Italy; 2 Venetian Institute of Molecular Medicine, 35100 Padua, Italy

**Keywords:** Aging, Oxidative stress, Longevity, Risk assessment

## Abstract

**Background:**

The risk of diabetic complications is modified by genetic and epigenetic factors. *p66Shc* drives the hyperglycaemic cell damage and its deletion prevents experimental diabetic complications. We herein tested whether *p66Shc* expression in peripheral blood mononuclear cells (PBMCs) predicts adverse outcomes in people with diabetes.

**Methods:**

In a cohort of 100 patients with diabetes (16 type 1 and 84 type 2), we quantified baseline *p66Shc* expression in PBMCs by quantitative PCR. Patients were extensively characterized for demographics, anthropometrics, biochemical data, prevalence of complications, and medications. With a pseudo-prospective design, we retrieved cardiovascular death, major adverse cardiovascular events (MACE), and new occurrence of micro- or macroangiopathy during follow-up.

**Results:**

At baseline, patients were on average 60 year old, with 10-year diabetes duration, and overall poor glycaemic control (HbA1c 7.8%). Patients with high versus low *p66Shc* expression (based on median value) had very similar baseline characteristics. Average *p66Shc* expression did not differ by presence/absence of complications. During a median 5.6-year follow-up, the primary endpoint of cardiovascular death or MACE occurred in 22 patients, but no relation was detected between cardiovascular outcomes and *p66Shc* expression. In patients who developed new complications at follow-up, baseline *p66Shc* was significantly higher, especially for macroangiopathy. The incidence of new macroangiopathy was > 3-times higher in patients with high versus those with low baseline *p66Shc* expression.

**Conclusions:**

*p66Shc* expression in PBMCs was not associated with prevalent diabetic complications but predicted new onset of complications, especially macroangiopathy, although no relation with hard cardiovascular endpoints was detected.

## Background

Development and progression of chronic diabetic complications are closely related to disease duration, glycaemic control, and concomitant risk factors. However, genetic and epigenetic factors likely modulate the risk of complications, because some patients develop complications despite good glycaemic control, and some others do not despite lifelong poor glycaemic control [1–3]. Understanding what drives such an individual predisposition is important to devise new therapeutic approaches to counter the diabetic complication burden.

In mammals, the 66 kDa isoform of the Shc gene product (*p66Shc*) differs from the other isoforms (p46 and p52) because it is phosphorylated on serine, translocates to the mitochondrial intermembrane space, and catalyses the production of hydrogen peroxide (H_2_O_2_), promoting cellular oxidative stress [4]. *p66Shc* is activated by hyperglycaemia mainly through protein kinase C [5], and is supposed to be one mediator of hyperglycaemic cell damage. Indeed, genetic deletion of *p66Shc* protects mice from several features of experimental diabetic complications, such as nephropathy [6], autonomic neuropathy [7], endothelial dysfunction [8], cardiomyopathy [9], and delayed wound healing [10]. The originally described *p66Shc*^−/−^ mice were long-lived in a controlled housing environment but severely counter-selected is a free-living setting, owing to impaired response to cold and stress [11, 12]. Together with the observations that *p66Shc* promotes adipogenesis [13], these notions identify *p66Shc* as a candidate thrifty gene.

Furthermore, p66Shc has been suggested to control vascular hyperglycaemic memory in diabetes [14], thereby representing one candidate modulator of the risk of complications.

In patients with diabetes, the expression of *p66Shc* is increased, as demonstrated in peripheral blood mononuclear cells (PBMCs) [15]. *p66Shc* upregulation in PBMC has also been shown in patients with acute coronary syndromes and stroke [16, 17]. Thus, the biological processes regulated by *p66Shc* in mice may also be active in humans.

To test this hypothesis in the setting of diabetes, we performed a longitudinal evaluation of patients having a baseline determination of *p66Shc* gene expression in PBMCs, who were followed-up to collect data on cardiovascular outcomes and progression of complications.

## Methods

### Study design

This was a pseudo-prospective study. Baseline data were recorded at time of *p66Shc* expression analysis, whereas follow-up data were collected retrospectively from August 2017 to baseline by accessing patients’ electronic files. The routine follow-up of patients at 6-month intervals and standardization of electronic chart records allowed simulating a prospective design.

### Recruitment and characterization of patients

Patients were recruited at the Diabetes Outpatient Clinic of the University Hospital of Padova between 2011 and 2012. Inclusion criteria were: a diabetes diagnosis according to ADA criteria, age 18–80, both genders. Exclusion criteria were: acute disease or infection; recent (within 3 months) surgery, trauma or cardiovascular event at baseline; immune disorders or organ transplantation; cancer; advanced liver (cirrhosis) or kidney (uremia) disease; pregnancy or lactation; inability to provide informed consent.

We collected the following baseline data for each patient: age, sex, BMI, diabetes duration, HbA1c, systolic and diastolic blood pressure, urinary albumin creatinine ratio (UACR), serum creatinine, lipid profile (total, LDL, and HDL cholesterol and triglycerides), and medications. The estimated glomerular filtration rate (eGFR) was calculated according to the CKD-EPI formula [18]. Hypertension was defined as a systolic blood pressure ≥ 140 mmHg or a diastolic blood pressure ≥ 90 mmHg, or the use of anti-hypertensive medications.

We recorded detailed information on diabetic complications, as follows. Retinopathy was defined on the basis of a digital funduscopic examination and scored remotely by an experienced ophthalmologist, according to the ETDR study [19]. Somatic neuropathy was defined in the presence of typical sensory or motor symptoms, confirmed by clinical examination (vibratory perception threshold and monofilament sensitivity) and eventual determination of neural conduction velocity. Autonomic neuropathy was screened annually using four routine cardiovascular autonomic function tests: deep breathing, lying-to-standing, Valsalva manoeuvre (Neurotester system) and orthostatic hypotension. Nephropathy was defined as either an albumin excretion rate of 30 mg/g creatinine or higher, or as an eGFR of 60 mL/min/1.73 mq or lower. Microangiopathy was defined as the presence of either retinopathy, neuropathy, or nephropathy or a combination thereof.

Coronary artery disease (CAD) was defined as a history of myocardial infarction or angina, or evidence of significant coronary artery disease at coronary angiography. Peripheral arterial disease (PAD) was defined as a history of claudication or rest pain, or significant stenosis in leg arteries. Cerebrovascular disease (CerVD) was defined as symptomatic or asymptomatic stenosis carotid arteries (at least 30% lumen narrowing) or the presence of a past history of stroke/transient ischemic attack. Macroangiopathy was defined as the presence of CAD, PAD, or CerVD, or a combination thereof. At baseline, all patients also underwent additional cardiovascular characterization by a carotid ultrasound with recording of maximal carotid intima-media thickness (IMT) and degree of stenosis, a determination of the ankle-brachial index (ABI), and an echocardiography with recording of systolic dysfunction (an ejection fraction of 50% or lower) and diastolic dysfunction (defined as E:A reversal).

### Baseline determination of *p66Shc* expression

PBMCs were collected from 20 mL of blood over Histopaque-1077 (Sigma-Aldrich, Milano, Italy) as follows. Blood (20 mL) was drawn into tubes containing EDTA, transferred to a 40-mL plastic centrifuge tube, diluted with 20 mL of phosphate buffer saline (PBS) and gently mixed. The diluted blood was gently layered on 5 mL of Ficoll-Paque in a centrifuge tube and centrifuged at 360 g for 50 min at room temperature; the supernatant was carefully aspirated and discarded. The mononuclear cells at the interface with the plasma were pipetted into a plastic centrifuge tube, washed with PBS, and centrifuged twice at 170 g for 10 min. The mononuclear fraction contained 90–93% lymphocytes and 3–5% PBMCs when evaluated by Wright staining.

Total RNA was extracted using RNeasy Mini Kit, (QIAGEN) after erythrocytes lysis. To eliminate genomic DNA contamination, 1 µg of total RNA was treated with DNase I, Amp Grade (Invitrogen, USA) before reverse transcription (RT). cDNA was then synthesized with the iScript cDNA synthesis kit (Bio-Rad, USA) according to the manufacturer’s instructions. Quantitative real-time polymerase chain reaction (qPCR) assay was performed in a Thermal Cycler CFX-96 (Biorad). The PCR reaction was performed in a 25 µL final reaction volume containing 200 nmol of each primer and 5X SYBR Green SuperMix (Bio-Rad, USA). All the reactions were performed in 96-well plates. A negative control containing all reagents but no cDNA template was included in all runs. Primers were designed from sequences derived from the GenBank database using Primer 3 (Whitehead Institute, Massachusetts, USA) and Operon’s Oligo software (Operon, California, USA) and were purchased from Eurofins MWG (Ebersberg, Germany). The *p66Shc* primers were sense 5′AATCAGAGAGCCTGCCACATT′3, antisense 3′CTCTTCCTCCTCCTCATC5′ (NM_001130040). Validation of specificity of qPCR assay was performed by melt-curve analysis and by agarose gel analysis. β-actin was used as the reference gene (primers sense 5′AGAGCTACGAGCTGCCTGAC′3, antisense 3′GGATGCCACAGGACTCCA5′; NM_001101.3). A calibration curve was generated with threshold cycle (Cq) values from serial dilutions of cDNA (from 106 to 10 copies/reaction) to determine reaction efficiencies, linearity, detection and quantification limits. Data analyses were performed with the iQ Optical System Software (Bio-Rad, Hercules, CA). The comparative cycle threshold method (∆∆Cq), which compares the between groups difference in cycle threshold values, was used to obtain the relative fold change of gene expression. Reproducibility of the assay was evaluated by a test–retest strategy in 16 duplicate cases: the coefficient of variation was 9.2% and the intraclass correlation coefficient was 0.93. Four patients had repeated measures, performed on average 3 months apart: intraclass correlation coefficient was 0.85, showing a relative stability of p66Shc expression over time.

### Follow-up and definition of events

The primary outcome was a first expanded MACE (major adverse cardiovascular event), defined as cardiovascular death, non-fatal acute myocardial infarction (AMI), non-fatal stroke, unstable angina, unplanned revascularization, or hospitalization for heart failure.

Event definition and adjudication was performed as in our previous pseudo-prospective studies [20]. The cause of death was determined by the principal condition and was considered to be cardiovascular in case of: sudden death; death occurring up to 14 days after an acute myocardial infarction; death occurring in the context of clinically worsening symptoms and/or signs of heart failure; death occurring up to 30 days after a stroke; death due to another documented cardiovascular cause (e.g. dysrhythmia, pulmonary embolism, or intervention). Any death not attributed to a non-cardiovascular cause were presumed to be cardiovascular. Nonfatal myocardial infarction was defined in the presence of at least 2 of the following 3 criteria: cardiac biomarker elevation; electrocardiographic changes consistent with new ischemia; imaging evidence of new non-viable myocardium or new wall motion abnormalities. Nonfatal stroke was defined as the rapid onset of a focal/global neurological deficit (change in level of consciousness, hemiplegia, hemiparesis, numbness or sensory loss affecting one side of the body; dysphasia/aphasia; hemianopia, other new neurological sign/symptom), with a duration of ≥ 24 h (< 24 h if the event was associated with pharmacologic treatment, or in the presence of available brain imaging showing new haemorrhage or infarct, or resulting in death), and confirmed by a neurology specialist or by brain imaging. Unstable angina was defined as resting, new onset, or worsening angina, in the absence of elevation in cardiac biomarkers, and in the presence of new or worsening ST-T changes on ECG, or evidence of ischemia by cardiac imaging, or angiographic evidence of ≥ 70% stenosis in an epicardial coronary artery. Heart failure was defined in the presence of typical clinical manifestations or their worsening (dyspnoea, orthopnoea, paroxysmal nocturnal dyspnoea, oedema, pulmonary basilar crackles, jugular venous distension, third heart sound or gallop rhythm, radiologic evidence of worsening heart failure), needing new therapy or up-titration of doses (diuretics, inotropes, vasodilators), eventually supported by changes in biomarkers (e.g. brain natriuretic peptides). Unplanned coronary, peripheral, or carotid revascularization was considered if occurred > 6 months after baseline.

The secondary outcome was development of new microangiopathy or macroangiopathy. New-onset macroangiopathy was defined as the new occurrence of CAD, PAD, or CerVD in patients who were free from any macroangiopathy at baseline. New-onset microangiopathy was defined as the new occurrence of retinopathy, neuropathy, or nephropathy in patients who were free from any microangiopathy at baseline.

### Sample size calculation

Based on the baseline clinical characteristics of patients, we assumed an annual rate of cardiovascular events and death (MACE) of at least 4%. Thus, with n = 100 patients and a follow-up of 5 years, we estimated that at least 20 MACE had to be recorded: the study had 80% power to detect a significant higher incidence of MACE in the group with above median *p66Shc* expression (n = 50) versus those with below median *p66Shc* expression (n = 50) if the true relative risk was > 3.0.

### Statistical analysis

Normality of continuous data was checked using the Kolmogorov–Smirnov test. Normal continuous variables are expressed as mean ± standard deviation and non-normal variables were log-transformed before analysis. Categorical variables are expressed as percentage. Comparisons in continuous variables between two groups were analysed using unpaired 2-tail Student’s t test, whereas comparisons in categorical variables were analysed with the Chi square test. The Bonferroni correction was used to adjust alpha for multiple testing. Correlations were checked using the Pearson’s r coefficient. To detect independent determinants of the outcome, we performed a multivariable logistic regression analysis, where a dichotomous endpoint was the dependent variable and confounders were entered as independent (explanatory) variables. SPSS version 23 or higher was used and statistical significance was accepted at p < 0.05.

## Results

### Characteristics of the study cohort

*p66Shc* gene expression was determined in peripheral blood mononuclear cells of 100 patients with diabetes. Clinical characteristics of the study population are reported in Table 1. We included 16 patients with type 1 diabetes and 84 with type 2 diabetes. Patients were about 60 years old, with an average diabetes duration of 10 years, and an overall poor glycaemic control (HbA1c 7.8%).

**Table 1:** Baseline clinical characteristics of study patients

Variable	All patients	Low *p66Shc* expression	High *p66Shc* expression	*p* value
Demographics				
Number	100	51	49	–
Age, (years)	61.8 ± 10.8	62.1 ± 12.1	61.4 ± 9.4	0.823
Sex male, (%)	60	55	65	0.293
Diabetes data				
Type 1/type 2, (%)	16/84	20/80	12/88	0.320
Diabetes duration, (years)	10.2 ± 8.0	10.4 ± 8.1	10.1 ± 7.9	0.866
HbA1c, % (mmol/mol)	7.8 ± 1.6 (62 ± 13)	8.0 ± 1.6 (64 ± 13)	7.7 ± 1.6 (61 ± 13)	0.376
Fasting plasma glucose, mg/dL (mmol/L)	173.5 ± 63.8 (9.6 ± 3.5)	180.3 ± 70.6 (10.0 ± 3.9)	166.7 ± 55.9 (9.3 ± 3.1)	0.293
Concomitant risk factors				
BMI, (kg/m^2^)	29.0 ± 4.7	29.4 ± 4.9	28.5 ± 4.6	0.377
Waist circumference, (cm)	102.4 ± 14.1	103.9 ± 13.6	100.9 ± 14.6	0.300
Current smoking, (%)	11	12	10	0.805
Hypertension, (%)	70	78	61	0.061
Systolic blood pressure, (mmHg)	137.4 ± 18.8	140.3 ± 18.0	134.5 ± 19.3	0.123
Diastolic blood pressure, (mmHg)	80.3 ± 9.5	82.0 ± 9.7	78.6 ± 9.0	0.073
Total cholesterol, mg/dL (mmol/L)	184.6 ± 38.3 (4.7 ± 1.0)	184.5 ± 35.9 (4.7 ± 0.9)	184.8 ± 41.0 (4.7 ± 1.1)	0.968
HDL cholesterol, mg/dL (mmol/L)	52.4 ± 16.7 (1.3 ± 0.4)	51.8 ± 15.0 (1.3 ± 0.4)	53.0 ± 18.7 (1.4 ± 0.5)	0.722
LDL cholesterol, mg/dL (mmol/L)	108.0 ± 32.0 (2.8 ± 0.8)	106.4 ± 29.2 (2.7 ± 0.7)	110.0 ± 35.0 (2.8 ± 0.9)	0.598
Triglycerides, mg/dL (mmol/L)	121.0 ± 59.9 (1.4 ± 0.7)	131.6 ± 67.6 (1.5 ± 0.8)	110.1 ± 49.0 (1.2 ± 0.6)	0.073
Macroangiopathy				
Coronary artery disease, (%)	14	16	12	0.624
Peripheral arterial disease, (%)	12	14	10	0.592
Ankle-brachial index	1.18 ± 0.20	1.18 ± 0.20	1.17 ± 0.19	0.705
Cerebrovascular disease, (%)	51	47	55	0.426
Max carotid IMT, mm	0.94 ± 0.23	0.95 ± 0.21	0.93 ± 0.25	0.725
Max carotid stenosis, (%)	15.1 ± 7.3	14.4 ± 17.8	15.9 ± 16.9	0.665
Cardiac systolic dysfunction, (%)	5	8	2	0.187
Cardiac diastolic dysfunction, (%)	35	33	27	0.463
At least one macroangiopathy, (%)	62	65	59	0.574
Microangiopathy				
Retinopathy, (%)	29	33	20	0.149
Autonomic neuropathy, (%)	12	12	12	0.942
Somatic neuropathy, (%)	28	22	35	0.147
Albumin-creatinine ratio, (mg/g)	80.3 ± 212.6	116.3 ± 289.4	43.5 ± 76.2	0.089
Albuminuria > 30 mg/g, (%)	37	39	33	0.537
Serum creatinine, (µmol/L)	81.7 ± 17.7	82.9 ± 20.7	80.4 ± 14.0	0.473
eGFR, mL/min/1.73 m^2^	82.2 ± 18.5	80.3 ± 19.7	84.1 ± 17.2	0.309
CKD stage III or higher, (%)	14	17	10	0.390
At least one microangiopathy, (%)	76	80	71	0.299
Glucose lowering therapies (%)				
Insulin	37	37	37	0.958
Metformin	63	63	63	0.958
Sulphonylurea	27	37	16	0.024*
Repaglinide	9	8	10	0.684
Thiazolidinedione	2	4	0	0.165
DPP-4 inhibitors	8	8	8	0.954
GLP-1 receptor agonist	1	0	2	0.310
Other therapies (%)				
ACE inhibitor/ARB	64	71	57	0.165
Other pressure lowering drugs	51	65	37	0.009*
Aspirin	45	49	41	0.415
Statin	56	61	51	0.330

Patients were divided into equal groups based on the median value of baseline *p66Shc* expression (low vs high)

* Not significant after Bonferroni correction

### Cross-sectional association between *p66Shc* expression and complications

We first divided patients according to low or high *p66Shc* gene expression in PBMCs (Table 1), based on the median value (1.17 relative expression [ΔΔ^Ct^]). We detected no differences in all clinical and biochemical variables between the two groups. Marginal differences in the use of some medications did not survive after Bonferroni correction.

**Fig. 1:**
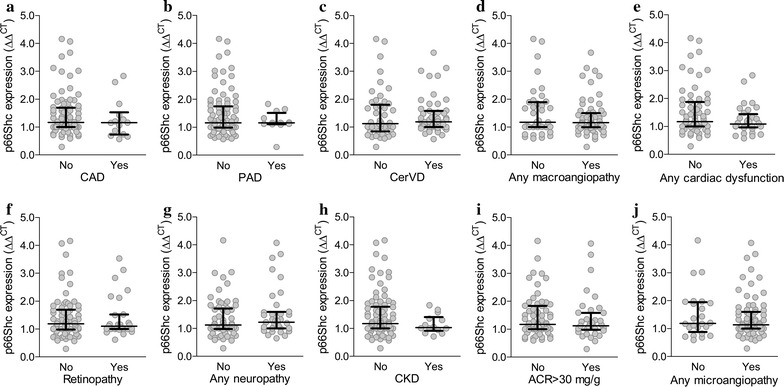
*p66Shc* gene expression in relation to baseline complications. Scatter plots show *p66Shc* expression in patients divided according to the presence or absence of diabetic macroangiopathy (**a**–**e**) or microangiopathy (**f**–**j**) at baseline. Median and interquartile range are superimposed to scattered data points

We then evaluated *p66Shc* expression according to the presence or absence of complications. We detected no significant differences in the average *p66Shc* expression between patients with and without coronary artery disease, peripheral arterial disease, cerebrovascular disease, any macroangiopathy, cardiac dysfunction, retinopathy, neuropathy, chronic kidney disease, micro/macroalbuminuria, and any microangiopathy (Fig. 1).

No linear correlation was detected between *p66Shc* expression and age, BMI, blood pressure, diabetes duration, measures of glucose control (HbA1c and fasting plasma glucose), lipids, and renal function.

Relative baseline expression of *p66Shc* in PBMCs was very similar in type 1 (1.43 ± 0.87) versus type 2 diabetes (1.41 ± 0.75; p = 0.934). Results of this cross-sectional analysis did not change by excluding patients with type 1 diabetes.

### Association between baseline *p66Shc* expression and cardiovascular outcomes

After the baseline determination of *p66Shc* gene expression, patients were followed-up for a median of 5.6 (IQR 5.0–6.4) years. Information on 11 patients could not be recalled; baseline clinical characteristics of patients lost to follow-up did not differ from those of patients with complete follow-up data (not shown). During this period, 6/89 patients died (3 for cancer, 2 for cardiovascular causes, and 1 for respiratory failure), 20/83 alive patients experienced at least one major adverse cardiovascular event (MACE), and 13 patients experienced more than one MACE. Among the 20 patients with incident MACE, the first event was acute myocardial infarction in 1, stroke or TIA in 4, unstable angina in 3, hospitalization for heart failure in 9, and unplanned revascularization in 4 (Table 2).

**Table 2:** Events recorded during follow-up

Outcome	Number of patients (%)
Complete follow-up	89 (100.0)
Death from any cause	6 (6.7)
Cardiovascular death	2 (2.2)
Cancer	3 (3.4)
Respiratory failure	1 (1.1)
First MACE	20 (22.4)
Acute myocardial infarction	1 (1.1)
Stroke/TIA	4 (4.5)
Unstable angina	3 (3.4)
Hospitalization for heart failure	8 (9.0)
Revascularization	4 (4.5)
Combined cardiovascular death or MACE	22 (24.7)
Patients with multiple MACE	13 (14.6)
Patients with new complications	22 (24.7)
New macroangiopathy	15 (16.9)
New coronary artery disease	6 (6.7)
New cerebrovascular disease	17 (19.1)
New peripheral arterial disease	15 (16.9)
New microangiopathy	11 (12.4)
New retinopathy	12 (13.5)
New nephropathy	10 (11.2)
New neuropathy	8 (9.0)

**Fig. 2:**
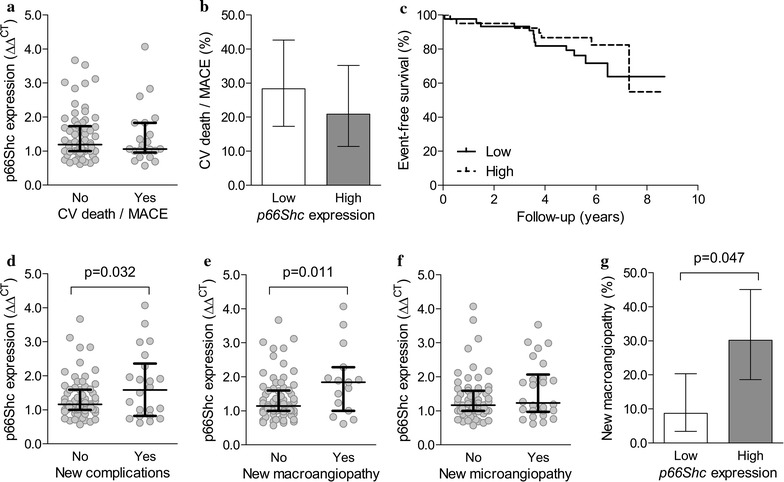
*p66Shc* gene expression in relation to future complications. **a** Average baseline *p66Shc* expression in patients with or without incident cardiovascular (CV) death or MACE during follow-up. **b** Incidence of CV death or MACE during follow-up in patients categorized in two groups according to the median value of baseline *p66Shc* expression. **c** Kaplan–Meier curves for CV death or MACE in patients with low versus those with high baseline *p66Shc* expression. **d**–**f** Average baseline *p66Shc* expression in patients with or without development of new complications (**d**), macroangiopathy (**e**), or microangiopathy (**f**) during follow-up. **g** Incidence of new macroangiopathy during follow-up in patients categorized in two groups according to the median value of baseline *p66Shc* expression. Scatter plots also report median and interquartile range

Gene expression of *p66Shc* did not differ significantly in patients with an adverse cardiovascular outcome (cardiovascular death and MACE, n = 22, annual rate of 4.4%) compared to those without (n = 77; Fig. 2a). The composite outcome of cardiovascular death or MACE occurred in 13 of 46 patients with low *p66Shc* expression and in 9 of 43 patients with high *p66Shc* expression (28.3% vs. 20.9%, p = 0.469; Fig. 2b, c).

### Association between baseline *p66Shc* expression and progression of complications

During follow-up, 22/89 patients (24.7%) developed new complications: 15 macroangiopathy, 11 microangiopathy, and 4 both. In patients who developed micro- or macroangiopathy, *p66Shc* expression was about 30% higher than in those who did not develop or already had baseline complications (p = 0.032; Fig. 2d). The association was significant for new macroangiopathy (p = 0.011; Fig. 2e), but not for new microangiopathy (p = 0.155; Fig. 2f). Also among patients who were free from macroangiopathy at baseline (n = 34/89), those who developed macroangiopathy had higher *p66Shc* expression than those who did not (1.24 ± 0.11 versus 1.87 ± 0.26; p = 0.023). New macroangiopathy occurred in 4/46 patients with low *p66Shc* expression and in 13/43 patients with high *p66Shc* expression (8.7% vs 30.2%; p = 0.047; Fig. 2g).

Among patients who were free from macroangiopathy at baseline, those who developed new macroangiopathy differed at p < 0.1 from those who did not only for higher baseline *p66Shc* expression (p = 0.023) and lower ABI (p = 0.088). Upon a multivariable analysis, the association between *p66Shc* expression and new development of macroangiopathy was independent from ABI (p = 0.048).

## Discussion

In this study, we found that diabetic patients with a high baseline gene expression of *p66Shc* in PBMCs experienced a > threefold increase in the risk of developing complications (especially macroangiopathy) over a 5-year period, than patients with lower *p66Shc* expression levels. This finding is in line with the strong evidence that *p66Shc* contributes to experimental diabetic complications, and suggests that *p66Shc* gene expression may be part of the epigenetic signature that modulates complication risk in humans [3].

Strikingly however, we detected almost no link between *p66Shc* gene expression and the prevalence of complications at baseline, despite we performed an extensive patient characterization. Our data contrast with a study showing increase *p66Shc* expression in PBMCs of patients with diabetic nephropathy and correlation with albumin excretion and disease duration [21]. Despite hyperglycaemia is known to activate *p66Shc* in vitro, we detected no correlation between *p66Shc* expression and HbA1c or fasting plasma glucose. In our study, patients with high versus those with low *p66Shc* expression were on average very similar at baseline.

Furthermore, we found no relation between *p66Shc* expression and hard cardiovascular endpoints, despite a link was noted with the development of macroangiopathy. The absolute number of MACE was relatively small, but a post hoc power calculation, given the trend we detected, suggested futility of expanding the study to detect a significant association between *p66Shc* expression and risk of MACE. The discrepancy between observed and expected results has unclear explanations, but likely indicate that *p66Shc* represents only one of the many genetic and epigenetic determinants of complications.

In this study, we included patients with type 1 or type 2 diabetes because preclinical studies have shown consistent results in the two models [7]. Furthermore, baseline *p66Shc* expression was very similar in type 1 versus type 2 diabetes. Nonetheless, the inclusion of both types of diabetes may have confounded cross-sectional associations, as the weight of glycemic control on the association with macrovascular complications is different type 1 versus type 2 diabetes. Therefore future studies should evaluate the associations between p66shc expression, cardiovascular outcomes and progression of complications separately in the two groups of patients.

One limitation of our study is that *p66Shc* expression was determined only in PBMCs. In clinical studies, it is difficult to have tissue samples from large cohorts of patients and, to our knowledge, studies on *p66Shc* have evaluated its expression only in blood cells [15–17], adipose tissue [22] and renal biopsies [21]. Although blood cells are actively involved in the pathogenesis of diabetic complications [23] and can be used as a surrogate to model pathophysiological processes in remote tissues, evaluation of metabolically active tissues or organs targeted by hyperglycaemic damage would be needed to better define the role of *p66Shc* in human diabetic complications.

The analysis of *p66Shc* expression was methodologically refined and showed good reproducibility and stability over time, but it was done only at baseline. Therefore, it will be useful in the future to test whether changes in expression reclassify the risk of adverse outcomes. Even more importantly, *p66Shc* activity is regulated by serine phosphorylation and mitochondrial translocation. Therefore, enzymatic activation may be more important than gene expression in determining the biological effects of *p66Shc*.

This is the largest clinical study on *p66Shc* expression in humans and the one with the longest follow-up. The rate of cardiovascular events and death is fairly consistent with that observed in cardiovascular outcome trials [24], but the number of patients with new complications or cardiovascular events was small in absolute terms. It is therefore possible that some findings did not reach statistical significance (e.g. association between baseline *p66Shc* expression and new microangiopathy) because of limited power. Therefore larger studies are needed to better dissect the relative contribution of p66Shc on the risk of macro- versus microangiopathy.

## Conclusions

We found that *p66Shc* expression in PBMC in not associated with prevalent diabetic complications but predicts future development of new complications over time. This was particularly evident for macroangiopathy, although baseline *p66Shc* expression did not predict hard cardiovascular outcomes. Although our study supports a role for *p66Shc* as an epigenetic modulator of diabetic complications, future studies should be larger, include an evaluation of *p66Shc* activity and, possibly, analyse expression in tissues directly targeted by the hyperglycaemic damage.

## Abbreviations

ABI: ankle-brachial index
ADA: American Diabetes Association
AMI: acute myocardial infarction
CAD: coronary artery disease
CerVD: cerebrovascular disease
CKD-EPI: Chronic kidney disease Epidemiology Collaboration
EDTA: ethylene diamino-acetic acid
eGFR: estimated glomerular filtration rate
ETDR: early treatment of diabetic retinopathy
IMT: intima-media thickness
IQR: interquartile range
MACE: major adverse cardiovascular events
PAD: peripheral arterial disease
PBMC: peripheral blood mononuclear cells
PBS: phosphate buffer saline
PCR: polymerase chair reaction
Shc: Src-homology collagen
UACR: urinary albumin creatinine ratio
